# Chylothorax due to hepatic alveolar echinococcosis with infiltration of diaphragm and left pleura: a case report

**DOI:** 10.1186/s12879-023-08180-5

**Published:** 2023-04-14

**Authors:** Anne Schneider, Steffen Klengel, Christoph Lübbert, Henning Trawinski

**Affiliations:** 1 Division of Infectious Diseases and Tropical Medicine, Department of Medicine I, Leipzig University Medical Center, Liebigstr. 20, 04103 Leipzig, Germany; 2 Interdisciplinary Center for Infectious Diseases, Leipzig University Medical Center, Leipzig, Germany; 3 Department of Radiology, Medical Service Center RadCom, I.B, Riesa, S.K Germany; 4 Department of Infectious Diseases/Tropical Medicine, St. Georg Hospital, Leipzig, Germany

**Keywords:** Echinococcosis, Alveolar, Fox tapeworm, *Echinococcus multilocularis*, chylothorax, Pleura

## Abstract

**Background:**

Alveolar echinococcosis (AE) is an endemic parasitic zoonosis in Germany. In most cases, the liver is the primary organ affected.

**Case presentation:**

A 59-year old female patient presented with increasing exertional dyspnea and unintentional weight loss. A computed tomography (CT) scan showed a left-sided chylous pleural effusion and multiple intrahepatic masses with infiltration of the diaphragm and the pleura. The findings were initially misinterpreted as hepatocellular carcinoma (HCC) with infiltrating growth. Liver biopsy of one of the masses showed no evidence of malignancy, but an amorphous necrosis of unclear origin. HCC was further ruled out by magnetic resonance imaging (MRI). However, MRI findings were highly suspicious for hepatothoracic dissemination and complications due to AE. Typical histologic findings in a repeated and more specific examination of the liver tissue and a positive serology for echinococcosis confirmed the diagnosis of AE. As the hepatic and pulmonary manifestations were considered inoperable in a curative matter, an anti-parasitic treatment with albendazole was initiated. A video-assisted thoracoscopic surgery (VATS) with removal of the chylous effusion as well as a talc pleurodesis was performed to relieve the patient from dyspnea. Two months later, the patient was asymptomatic and a positron emission tomography (PET)-CT-scan with [18 F] fluoro-2-deoxy-d-glucose (FDG) showed a remarkable diminution of the hepatic manifestation.

**Conclusions:**

This case demonstrates a rare presentation of alveolar echinococcosis with a focus on pulmonary symptoms, emphasizing the importance of evaluation for pulmonary involvement in patients with AE and respiratory symptoms.

## Background

Echinococcosis is a parasitic zoonotic disease in humans. It is caused by the cestodes *Echinococcus granulosus sensu lato (s.l.)* leading to cystic echinococcosis (CE), also known as hydatid disease, and *Echinococcus multilocularis* leading to alveolar echinococcosis (AE). The incubation period may take up to several years in both forms.

CE is a zoonotic disease found in many countries worldwide. In Europe, autochthonous cases are found in the Mediterranean and in Eastern Europe, especially in the Balkan states. The disease is not endemic in Germany and usually considered imported [1]. It is transmitted by dogs who get infected by the ingestion of organs of sheep, cattle or pigs (intermediate hosts) containing hydatid cysts. Inside their final host, the larvae of *E. granulosus s.l.* develop to adult tapeworms of 3–7 millimeters in length. Infective worm eggs are then excreted and spread with the dog’s feces. As the eggs are very resistant, they may survive in the environment up to several years. Humans may become infected with *Echinococcus spp.* following ingestion or indirect contact with contaminated water, soil or food, leading to growth of hydatid cysts within the liver (70%), the lung (20%) or other organs as brain, spleen, kidneys and heart [2]. While many patients remain asymptomatic, symptoms such as abdominal pain, poor appetite, vomiting, icterus, chest pain or dyspnea may occur depending on size and location of the cysts.

AE is only found in countries across the northern hemisphere. In Europe it is endemic in Austria, France, Switzerland, Czech Republic and Germany with a widening of the geographical distribution during the last decades [3]. 91% of the approximately 18.200 new cases per year globally occur in China [2]. The disease is also endemic in Central Asia and Northern America. Foxes, which represent the main definitive hosts, get infected by the ingestion of rodents (intermediate hosts) containing larvae of *E. multilocularis*. Similar to the transmission of CE, eggs are shed into the environment with the final host’s feces and may eventually infect humans by the accidental ingestion of contaminated material. The tapeworm larvae primarily develop within the liver, although other organs may be affected through hematogenous or lymphogenous spread. While CE leads to the formation of cystic space-occupying lesions, AE shows a progressive, tumor-like growth with potential infiltration of all surrounding structures. Symptoms are usually abdominal pain or cholestasis with or without jaundice and depend on location and size of the lesion. If left untreated, AE may result in severe complications as billiary cirrhosis, hepatic failure and septic shock due to secondary bacterial infection [4]. Drinking water from natural sources and eating unwashed or uncooked vegetables, mushrooms or berries from fields and forests were not shown to be significant risk factors for AE in a German cohort of 40 cases, while being a farmer, chewing grass and owning a game hunting dog increased the risk for the disease significantly [5].

Diagnosis of echinococcosis is based on symptoms, epidemiological criteria, typical findings in ultrasound, CT-scan or MRI, and a positive serology (enzyme-linked immunoassay [ELISA], immunoblot, indirect haemagglutination [IHA]) [6]. A serological differentiation between AE and CE is not always possible due to cross-reactions. Furthermore, serology may be false negative, especially in immunosuppressed patients [2, 7]. Diagnostic biopsies of cystic lesions in CE pose a risk of parasite dissemination and should therefore only be considered in unclear cases in combination with diagnostic nucleic acid amplification testing (NAAT) or specific immunostaining [2]. If AE is suspected and serology remains negative, diagnosis should be forced by biopsy and histopathological examination and/or NAAT.

Antiparasitic treatment options include the benzimidazoles albendazole or mebendazole. Especially in AE, radical curative surgical removal of the cysts should be performed, followed by at least two years of benzimidazoles therapy. In inoperable cases, lifelong antiparasitic treatment is usually necessary [2, 6].

## Case presentation

In February 2021, a 59-year old female patient from Saxony, Germany, presented to a community hospital due to persisting exertional dyspnea (class II – III, according to New York Heart Association [NYHA]). She had suffered from a mild course of COVID-19 four months earlier. The otherwise healthy patient additionally reported an unintentional weight loss of 8 kg within the last 3 months. She declined fever or night sweats. Travel history included visits to the People’s Republic of China in 2015 and Iran in 2017, but no recent stays outside of Germany was reported. The patient declined regular contact to domestic animals; however, due to the close proximity of her home to a forest, foxes regularly appeared in her surroundings.

A chest X-ray showed a large left-sided pleural effusion. By means of thoracocentesis, several liters of a cloudy white exudate were evacuated. Histopathologic and microbiologic examination of the exudate indicated the presence of chylous effusion without signs of malignancy, bacterial or fungal infection. Unfortunately, further biochemical analysis of the pleural fluid with examination for lipid components has not been carried out. Additionally, a specific interferon-gamma release assay (IGRA) for tuberculosis was negative.

**Fig. 1:**
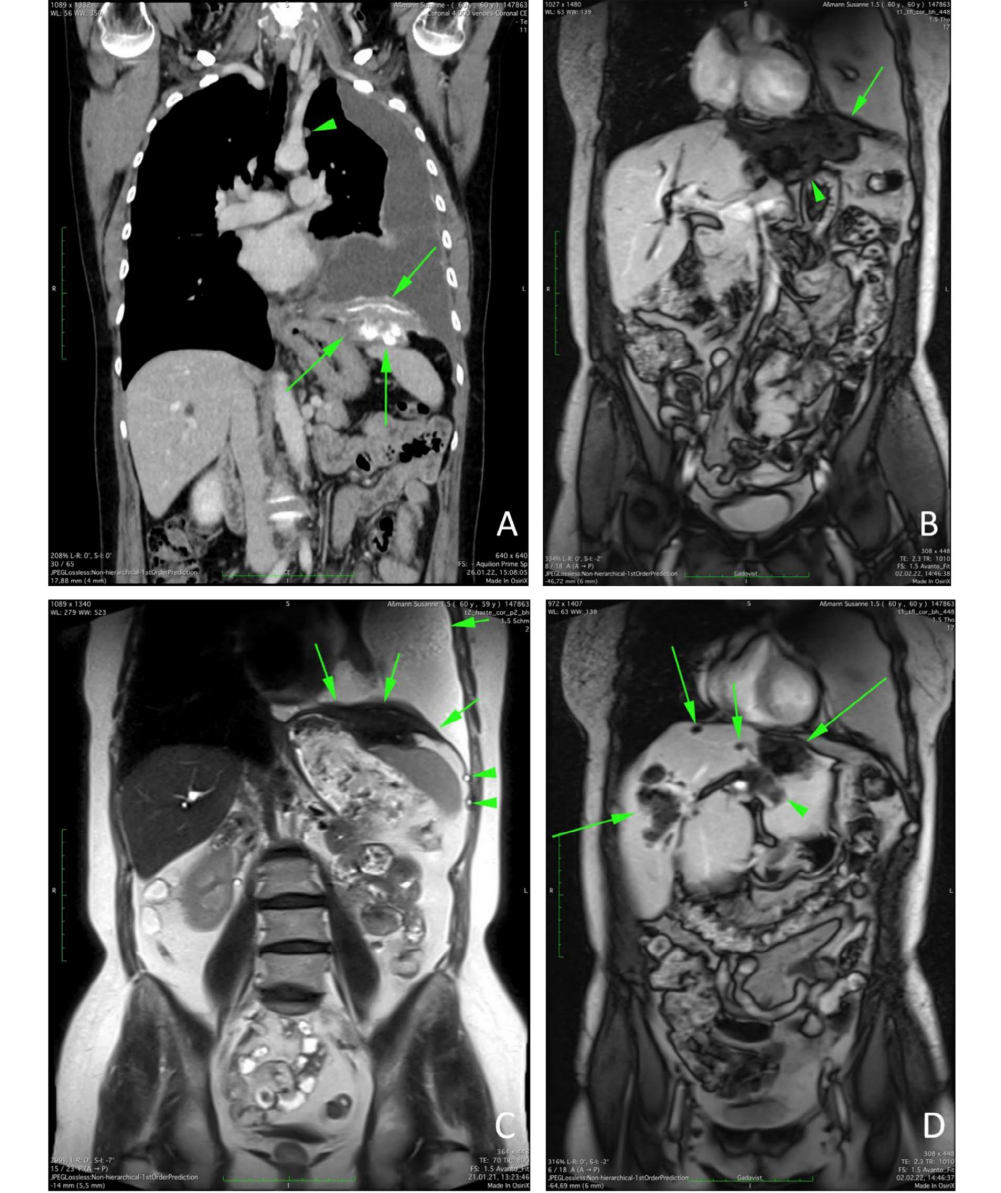
A: CT scan, post contrast, coronal plane. Green arrow: fibrotic and calcified infiltration of the left diaphragm and the spleen and pleural effusion left due to *Echinococcus multilocularis*. Green triangle: echinococcal cyst in the left pleura. B: MRI scan, T1 weighted, late phase, post contrast, coronal plane. Green arrow and triangle: per continuitatem inflammatory hypovascular/ fibrotic infiltration due to *Echinococccus multilocularis* from the liver towards the left diaphragm. C: MRI scan, T2 weighted, coronal plane. Green arrows: fibrotic infiltration of the left diaphragm and the spleen with pleural effusion left due to *Echinococcus multilocularis*. Green triangle: small echinococcal cyst in the left pleura. D: MRI scan, T1 weighted, late phase post contrast, coronal plane. Green arrows: confluent echinococcal cysts and confluent hypovascular fibrotic infiltrations in both liver lobes. Green triangle: perihilar fibrotic inflammatory infiltration.

A CT scan (Figure 1A) was initially misinterpreted as multifocal HCC infiltrating the left diaphragm, spleen, stomach and a loco-regional lymphadenopathy. While there was no histological evidence of malignancy, results of the liver biopsy were in line with low-grade drug-induced liver injury and an amorphous necrosis with surrounding tissue inflammation. Following MRI of the abdomen, a per continuitatem hepatothoracal spread (Figure 1B) with infiltration of the left diaphragm and the spleen (Figure 1C), based on AE cysts in both liver lobes (Figure 1D), was suspected. This led to a repeated liver biopsy, where histological examination showed an amorphous necrosis containing periodic acid-Schiff (PAS) positive, amorphous structures, as well as cystic structures, both indicating echinococcosis. Polymerase chain reaction (PCR) from liver tissue was negative for *Echinococcus* DNA, however, *Echinococcus* serology was positive indicating AE (*E. granulosus* and *E. multilocularis* screening tests positive, *Echinococcus* antigen B ELISA negative, *E. multilocularis* specific Em2 ELISA positive).

Lastly, the diagnosis of AE was made based on epidemiological history, imaging showing infiltrating growth of the lesion, histology and serology results [2, 6]. Treatment with albendazole 400 mg BID was started. The hepatic and pulmonary manifestations were considered inoperable by the members of the internal multidisciplinary tumor board. An infiltration of the thoracic duct was shown in an additional magnetic resonance lymphography (MRL) to be the underlying cause of the chylous pleural effusion. To relieve the patient from persistent dyspnea, a video-assisted thoracoscopic surgery (VATS) with evacuation of the exudate, pleurectomy, decortication and talc pleurodesis were performed. Diagnosis was confirmed by PCR from pleural biopsy being positive for *E. multilocularis* DNA. The surgical procedures releaved the patient of her dyspnea. FDG-PET scan combined with computed tomography (PET/CT scan) performed a few days postoperatively demonstrated a significant diminution of the pleural effusion with persisting elevated glucose metabolism along the pleura.

Following two months of treatment with albendazole, a repeat PET/CT scan was performed, which showed an almost complete reduction of glucose metabolism within the liver.

Unfortunately, the left-sided pleural effusion re-accumulated after some month. Nevertheless, we decided against further interventional procedure, as the patient remained oligo-symptomatic under continued pharmacotherapeutic treatment. Should the patient develop respiratory symptoms in the future, radiological intervention with coiling of the thoracic duct would be an option.

## Discussion and conclusion

We presented an uncommon case of a patient with dyspnea and weight-loss from Saxony, Germany, with hepatic AE resulting in multifocal confluent inflammatory fibrotic infiltrations of the liver, which spread per continuitatem trans-diaphragmatically to the left pleura, causing chylous pleural effusion due to an infiltration and compression of the thoracic duct. To our knowledge, this is the only published case of pulmonary AE with a chylous pleural effusion.

In CE, lungs are involved in around 20% (pulmonary hydatid disease). Hydatid cysts may rupture into the pleural space leading to pleural thickening and effusion, pneumothorax and empyema [8].The liver is the primary organ involved in most cases of AE [9], with most lesions located in the right liver lobe [6]. Local growth and infiltration, as well as hematogenous spread, may result in the involvement of other tissues and organs (e.g. diaphragm, lung, brain). According to surveillance data from the European Echinococcosis Registry collected from 1982 to 2000, 34% of AE patients are affected by extrahepatic spread [9]. Continuous growth most frequently affected the diaphragm, kidneys, adrenal glands, lungs or pleura [9]. Metastatic spread mainly affected the lung, brain or spleen [9]. Lungs are involved in 7–20% of cases of hepatic AE, and a large proportion of patients with pulmonary involvement are asymptomatic [10]. The presence of distant extrahepatic AE involvement is significantly related to the size of hepatic lesions [11].

In a recent retrospective case series, pulmonary involvement was reported in 34 of 261 hepatic AE cases (13%) [10]. An exclusive trans-diaphragmatic invasion of the lung was described in three patients (8,8%). In most cases, pulmonary involvement was due to hematogenous (73,5%) or a combined hematogenous and trans-diaphragmatic (17,7%) dissemination. An isolated involvement of the left lung was reported in three patients only. Similarly, pleural effusion was noted in only three patients, although there was a common relationship between AE lesions and the pleura on CT scan (73,5%). The majority of patients suffered from respiratory symptoms (dyspnea, thoracic pain, cough, hemoptysis). Dyspnea and weight loss were also the presenting symptoms of our patient. In the published case series [6], treatment consisted of antiparasitic treatment with albendazole in most cases. Only five patients underwent surgical treatment (wedge resection) of pulmonary lesions. As in our case, most patients with echinococcosis and pulmonary involvement are not deemed suitable for surgical intervention [10].

In conclusion, every patient with hepatic AE should be evaluated for pulmonary involvement by imaging, and respiratory symptoms and pulmonary or pleural lesions can be the presenting signs of AE. Furthermore, multiparametric MRI can provide relevant additional information that can support the diagnosis of AE.

## Data Availability

The datasets generated and analyzed during the current study are not publicly available as they contain sensitive personal data, but are available from the corresponding author on reasonable request.

## List of Abbreviations

AE: Alveolar echinococcosis
BID: Bis in die
CE: Cystic echinococcosis
CT: Computed tomography
DNA: Deoxyribonucleic acid
ELISA: Enzyme-linked immunoassay
FDG-PET: [18 F] fluoro-2-deoxy-d-glucose (FDG) Positron-Emission-Tomography
HCC: Hepatocellular carcinoma
IGRA: Interferon-gamma release assay
IHA: Indirect haemagglutination
MRI: Magnetic resonance imaging
MRL: Magnetic resonance lymphography
NAAT: Nucleic acid amplification testing
PAS: Periodic acid-Schiff
PCR: Polymerase chain reaction
VATS: Video-assisted thoracoscopic surgery
